# Carcinoembryonic Antigen Messenger RNA in Pelvic Lavage Fluid Predicts Local Recurrence After Curative‐Intent Resection of Rectal Cancer

**DOI:** 10.1002/ags3.70257

**Published:** 2026-07-31

**Authors:** Hiroyuki Asai, Yushi Yamakawa, Kazuyoshi Shiga, Shuhei Uehara, Akira Kato, Takuya Suzuki, Hajime Ushigome, Hiroki Takahashi, Yoichi Matsuo, Shuji Takiguchi

**Affiliations:** ^1^ Department of Gastroenterological Surgery Nagoya City University Graduate School of Medical Sciences Nagoya Aichi Japan; ^2^ Department of Gastroenterological Surgery Nagoya City University Hospital East Medical Center Nagoya Aichi Japan; ^3^ Department of Gastroenterological Surgery Nagoya City University Hospital West Medical Center Nagoya Aichi Japan

**Keywords:** CEA mRNA, local recurrence, pelvic lavage fluid, qRT‐PCR, rectal cancer

## Abstract

**Purpose:**

To evaluate whether pelvic lavage carcinoembryonic antigen (CEA) mRNA is associated with local recurrence after curative‐intent resection for rectal cancer and whether it provides additional prognostic information beyond circumferential resection margin (CRM) status.

**Methods:**

We retrospectively analyzed 178 patients who underwent curative‐intent resection for primary rectal cancer. Pelvic lavage fluid was collected intraoperatively before enterotomy, and CEA mRNA levels were quantified using quantitative reverse‐transcription polymerase chain reaction (qRT‐PCR). CEA mRNA positivity was defined as ≥ 0.26, and CRM positivity as ≤ 1 mm. Local recurrence was evaluated using Kaplan–Meier analysis and Cox proportional hazards modeling.

**Results:**

During a median follow‐up of 59.1 months, local recurrence occurred in 8 patients (4.5%). CEA mRNA was positive in 7 patients (3.9%), and CRM was positive in 11 patients (6.2%). Local recurrence rates were 36.4% (4/11) in CRM‐positive patients and 42.9% (3/7) in CEA mRNA–positive patients. All patients who tested positive for both markers (3/3) developed local recurrence, whereas the rate was only 2.5% (4/163) among those who tested negative for both markers. In multivariable analysis, CRM positivity (HR 11.09, 95% CI 2.06–59.68, *p* = 0.005) and CEA mRNA positivity (HR 5.99, 95% CI 1.06–33.81, *p* = 0.043) remained associated with local recurrence.

**Conclusions:**

Pelvic lavage CEA mRNA levels were associated with local recurrence beyond CRM status and may improve risk stratification. Further validation in larger multicenter cohorts is warranted.

## Graphical abstract

Pelvic lavage carcinoembryonic antigen (CEA) mRNA was quantified in 178 patients undergoing curative‐intent resection for rectal cancer. Positive pelvic lavage CEA mRNA was significantly associated with local recurrence and provided additional prognostic information beyond circumferential resection margin (CRM) status. Pelvic lavage CEA mRNA may help identify patients at high risk of local recurrence who could benefit from intensified postoperative surveillance.

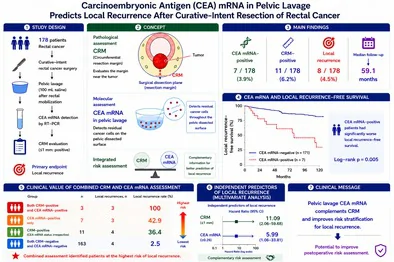

## 1 Introduction

Local recurrence after curative‐intent resection of rectal cancer remains a clinically important problem, comparable to distant metastasis, in terms of its impact on prognosis. However, unlike distant metastases, local recurrence is thought to arise from residual tumor cells located beyond the intended surgical dissection plane. Therefore, securing an appropriate surgical dissection plane within the confined pelvic cavity is essential for preventing local recurrence. Despite the substantial improvements in local control achieved through the introduction of total mesorectal excision (TME) and advances in multimodal treatments, local recurrence remains clinically relevant. Moreover, local recurrence is often difficult to treat and can substantially impair the quality of life (QOL) [1, 2].

Circumferential resection margins (CRM) are widely used pathological predictors of local recurrence. Several studies have reported that CRM involvement, typically defined as a margin distance of ≤ 1 mm, is associated with an increased risk of local recurrence and poor oncologic outcomes [3, 4, 5]. Nevertheless, CRM assessment depends on pathological processing and measurement techniques, which may vary across institutions. In addition, CRM does not directly reflect the presence of free or shed tumor cells that may disseminate from the surgical dissection plane during surgery, nor does it capture the overall status of the entire dissection plane.

In this context, the quantification of carcinoembryonic antigen (CEA) mRNA using real‐time quantitative RT‐PCR (qRT‐PCR) has attracted attention as a sensitive method for detecting small numbers of free tumor cells [6]. In gastric and colorectal cancers, CEA mRNA positivity in peritoneal lavage fluid has been reported to be associated with higher recurrence rates, even among cytology‐negative patients, and serves as an independent prognostic factor [7, 8, 9, 10, 11, 12, 13]. However, most of these studies were conducted primarily in open surgery cohorts, and although some included laparoscopic surgery, evidence in robot‐assisted surgery remains limited.

Minimally invasive surgery has become increasingly common in rectal cancer, and robot‐assisted surgery is expanding because it enables precise manipulation of the deep pelvis [14, 15, 16]. Against this background, we aimed to evaluate whether intraoperative CEA mRNA levels in pelvic lavage fluid predict local recurrence after curative‐intent resection for rectal cancer, and to assess its complementary value in combination with CRM status, including in a cohort undergoing minimally invasive approaches such as robot‐assisted surgery.

## 2 Methods

### 2.1 Patients

We retrospectively analyzed 178 patients who underwent curative‐intent resection for primary rectal cancer, including open, laparoscopic, and robot‐assisted surgery, at a single institution between April 2013 and November 2023.

Curative‐intent resection was defined as complete macroscopic tumor removal with R0 resection of the primary tumor.

The exclusion criteria were synchronous peritoneal dissemination, synchronous multiple colorectal cancers, and double cancers. Written informed consent was obtained from all the enrolled patients.

### 2.2 Collection of Pelvic Lavage Fluid

After sufficient mobilization of the rectum to the distal side and before enterotomy, 100 mL of normal saline was instilled into the pelvic cavity, gently agitated, and aspirated into sterile tubes. Enterotomy was performed only after the collection of lavage fluid to avoid exposure of the intestinal mucosa. The lavage fluid was then centrifuged at 2000 rpm for 10 min. After removal of the supernatant, the cell pellet was washed five times with heparinized normal saline, resuspended in 600 μL of RNeasy lysis buffer (Qiagen, Hilden, Germany) and RNA extraction buffer, and stored at −80°C until analysis.

### 2.3 RNA Extraction and cDNA Synthesis

After thawing, the total RNA was extracted using the RNeasy Plus Mini Kit (Qiagen) according to the manufacturer's protocol. Reverse transcription was performed using SuperScript III (Invitrogen, Carlsbad, CA, USA).

### 2.4 Quantification of CEA mRNA by qRT‐PCR

CEA mRNA levels were quantified by qRT‐PCR using CEA‐specific primers and a fluorescent probe on a LightCycler system (Roche Diagnostics). Glyceraldehyde‐3‐phosphate dehydrogenase (GAPDH) was used as an internal control. Based on receiver operating characteristic (ROC) curve analysis, a CEA mRNA level ≥ 0.26 was defined as positive.

### 2.5 Pathological Evaluation

The resected specimens were fixed in formalin, embedded in paraffin, and evaluated using standard pathological procedures to determine the pathological T category and lymph node metastasis. CRM was defined as the shortest distance from the tumor to the CRM, and CRM positivity was defined as ≤ 1 mm. The pathological evaluations were performed by a dedicated pathologist.

### 2.6 Definition of Local Recurrence and Follow‐Up

Local recurrence was defined as recurrence within the pelvic cavity below the sacral promontory and/or at the anastomotic site, excluding apparent lateral pelvic lymph node metastases. Recurrence was assessed using periodic computed tomography, serum CEA measurements, and clinical evaluation.

### 2.7 Statistical Analysis

All statistical analyses were performed using EZR software (Saitama Medical Center, Jichi Medical University). Statistical significance was defined as a two‐sided *p*‐value < 0.05.

The cut‐off value for pelvic lavage CEA mRNA to predict local recurrence was determined using receiver operating characteristic (ROC) curve analysis. The area under the ROC curve (AUC) and the 95% confidence interval (CI) were calculated using the DeLong method. The cut‐off value was selected based on the Youden index (sensitivity + specificity −1), which maximizes the sum of sensitivity and specificity. Internal validation was performed using bootstrap resampling (1000 iterations) to assess the stability of the AUC estimate.

Categorical variables were compared using Fisher's exact test. Local recurrence‐free survival was estimated using the Kaplan–Meier method and compared using the log‐rank test. Time to local recurrence was used as the outcome, and a Cox proportional hazards model was used for multivariate analysis, with CRM status (positive/negative) and CEA mRNA status (positive/negative) as covariates. Time zero was the date of surgery, and patients without local recurrence were censored at the last follow‐up.

## 3 Results

### 3.1 Patient Characteristics

In total, 178 patients were included in this study. The median follow‐up was 59.1 months (range, 0.7–137.9 months). The median age was 69 years (range, 30–93 years); 116 patients were male (65.2%), and 62 were female (34.8%). The tumor location was Rs in 56 patients (31.5%), Ra in 40 patients (22.5%), and Rb in 82 patients (46.1%). The pathological T category was T1 in 25 (14.0%), T2 in 41 (23.0%), T3 in 88 (49.4%), and T4 in 24 (13.5%) patients. Lymph node metastases were present in 68 patients (38.2%) and absent in 110 patients (61.8%). The pathological stage was I in 49 (27.5%) patients, II in 60 (33.7%), III in 59 (33.1%), and IV in 10 (5.6%). Among the 10 patients with Stage IV disease, 5 had liver metastases and 5 had lung metastases. Metastatic lesions were resected in 8 patients, whereas 2 patients underwent primary tumor resection without resection of metastatic lesions. The surgical approach was open in 13 (7.3%), laparoscopic in 99 (55.6%), and robot‐assisted in 66 (37.1%) patients. A total of 17 patients (9.6%) underwent neoadjuvant chemoradiotherapy (CRT), and 7 patients (3.9%) received neoadjuvant chemotherapy (NAC). In addition, 37 patients (20.8%) underwent lateral pelvic lymph node dissection (LLND) (Table 1).

**TABLE 1:** Characteristics of all patients (*N* = 178).

Variable	Category	*n*	%
Age, years—median (range)		69 (30–93)	—
Sex	Male/female	116/62	65.2/34.8
Tumor location	Rs/Ra/Rb	56/40/82	31.5/22.5/46.1
Pathological T category	T1/T2/T3/T4	25/41/88/24	14.0/23.0/49.4/13.5
Lymph node metastasis	Positive/negative	68/110	38.2/61.8
Pathological stage	I/II/III/IV	49/60/59/10	27.5/33.7/33.1/5.6
Surgical approach	Open/laparoscopic/robot‐assisted	13/99/66	7.3/55.6/37.1
Neoadjuvant treatment	CRT/NAC/TNT/none	17/7/0/154	9.6/3.9/0/86.5
Lateral pelvic lymph node dissection	Yes/no	37/141	20.8/79.2

### 3.2 Distribution of CEA mRNA Levels and Positivity Rate

CEA mRNA levels ranged from 0 to 2.77. ROC curve analysis yielded an AUC of 0.648 (95% confidence interval [CI], 0.450–0.846) for predicting local recurrence. The optimal cut‐off value determined by the Youden index was 0.26, corresponding to a sensitivity of 37.5% and specificity of 97.7% (Figure 1). Bootstrap internal validation (1000 resamples) showed a mean AUC of 0.646 (2.5th–97.5th percentile, 0.441–0.851). CEA mRNA was positive in 7 patients (3.9%). Among CEA mRNA–positive patients, six were male (85.7%); the tumor location was Ra in three (42.9%) and Rb in three (42.9%). Five patients (71.4%) had T3 disease, and 5 (71.4%) had lymph node metastasis. The pathological stage was II in 2 (28.6%) and III in 5 (71.4%) patients. The surgical approach was open in 3 patients (42.9%), laparoscopic in 3 (42.9%), and robot‐assisted in 1 (14.3%). Only 1 patient (14.3%) had undergone neoadjuvant chemoradiotherapy, whereas no patient had received neoadjuvant chemotherapy (Table 2). Among the 7 CEA mRNA–positive patients, local recurrence occurred in 3 (42.9%), whereas distant recurrence occurred in 2 (28.6%).

**FIGURE 1:**
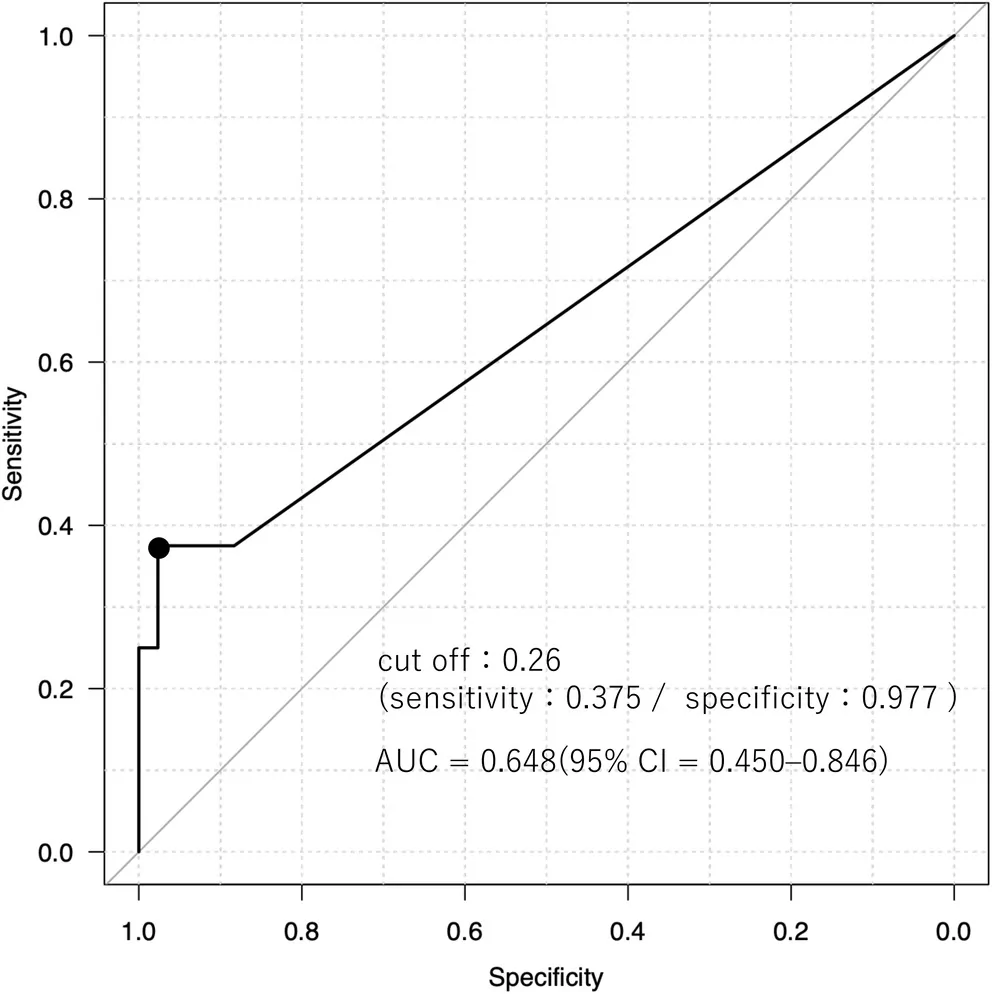
Receiver operating characteristic curve analysis for CEA mRNA in predicting local recurrence. The optimal cut‐off value for CEA mRNA positivity was 0.26, with a sensitivity of 37.5%, specificity of 97.7%, and an area under the curve of 0.648.

**TABLE 2:** Characteristics of patients with CEA mRNA–positive results (*N* = 7).

Variable	Category	*n*	%
Age, years—median (range)		67 (41–78)	—
Sex	Male/female	6/1	85.7/14.3
Tumor location	Rs/Ra/Rb	1/3/3	14.3/42.9/42.9
Pathological T category	T1/T2/T3/T4	0/0/5/2	0.0/0.0/71.4/28.6
Lymph node metastasis	Positive/negative	5/2	71.4/28.6
Pathological stage	I/II/III/IV	0/2/5/0	0.0/28.6/71.4/0.0
Surgical approach	Open/laparoscopic/robot‐assisted	3/3/1	42.9/42.9/14.3
Neoadjuvant treatment	CRT/NAC/TNT/none	1/0/0/6	14.3/0/0/85.7
Lateral pelvic lymph node dissection	Yes/no	1/6	14.3/85.7

### 3.3 CRM Positivity Rate

CRM was positive (≤ 1 mm) in 11 patients (6.2%). The median age was 66 years (range, 41–74 years), and 72.7% of patients were male. The tumors were predominantly Rb (72.7%). All CRM‐positive patients had an advanced pathological T category (T3 in 7 [63.6%] and T4 in 4 [36.4%]). Lymph node metastasis was observed in 6 patients (54.5%). The pathological stages ranged from II to IV, and there were no stage I cases. The laparoscopic approach was the most frequently used (54.5%). Two patients (18.2%) had undergone neoadjuvant chemoradiotherapy, whereas no patient had received neoadjuvant chemotherapy (Table 3).

**TABLE 3:** Characteristics of patients with CRM‐positive results (*N* = 11).

Variable	Category	*n*	%
Age, years—median (range)		66 (41–74)	—
Sex	Male/female	8/3	72.7/27.3
Tumor location	Rs/Ra/Rb	1/2/8	9.1/18.2/72.7
Pathological T category	T1/T2/T3/T4	0/0/7/4	0.0/0.0/63.6/36.4
Lymph node metastasis	Positive/negative	6/5	54.5/45.5
Pathological stage	I/II/III/IV	0/4/6/1	0.0/36.4/54.5/9.1
Surgical approach	Open/laparoscopic/robot‐assisted	2/6/3	18.2/54.5/27.3
Neoadjuvant treatment	CRT/NAC/TNT/none	2/0/0/9	18.2/0/0/81.8
Lateral pelvic lymph node dissection	Yes/no	6/5	54.5/45.5

### 3.4 Local Recurrence Rates

Local recurrence occurred in 8 of 178 patients (4.5%). Local recurrence rates were 36.4% (4/11) in CRM‐positive patients and 42.9% (3/7) in CEA mRNA–positive patients. All patients who were positive for both CRM and CEA mRNA (3/3) developed local recurrence, whereas the rate was 2.5% (4/163) among those negative for both markers.

### 3.5 Association Between CEA mRNA Status and Clinicopathological Factors

Associations between CEA mRNA levels and clinicopathological factors are shown in Table 4. Fisher's exact test showed that CEA mRNA positivity was significantly associated with advanced pathological T categories (pT3–4; *p* = 0.047), CRM positivity (*p* < 0.001), and open surgery (*p* = 0.009). There were no significant associations with age, sex, tumor location, lymph node metastasis, or lateral pelvic lymph node dissection.

**TABLE 4:** Association between CEA mRNA expression and clinicopathological factors.

Variable	Category	CEA mRNA‐positive (*n* = 7)	CEA mRNA‐negative (*n* = 171)	*p*
Age, years	≥ 75	2	42	0.681
	< 75	5	129	
Sex	Male	6	109	0.424
	Female	1	62	
Tumor location	Rb	3	80	1
	Other	4	91	
Pathological T category	≥ T3	7	105	0.047
	< T3	0	66	
Lymph node metastasis	Present	5	58	0.099
	Absent	2	113	
CRM status	Positive	3	8	< 0.001
	Negative	4	163	
Surgical approach	Open	3	10	0.009
	Laparoscopic or robot‐assisted	4	161	
Lateral pelvic lymph node dissection	Yes	1	36	1
	No	6	135	

### 3.6 Association Between Local Recurrence and Clinicopathological Factors

Associations between local recurrence and clinicopathological factors are shown in Table 5. Fisher's exact test revealed that advanced pathological T categories (pT3–4; *p* = 0.027), CEA mRNA positivity (*p* = 0.002), CRM positivity (*p* < 0.001), and positivity for both CEA mRNA and CRM (*p* < 0.001) were significantly associated with local recurrence.

**TABLE 5:** Association between local recurrence and clinicopathological factors.

Variable	Category	Local recurrence (*n* = 8)	No local recurrence (*n* = 170)	*p*
Age, years	≥ 75	0	44	0.203
	< 75	8	126	
Sex	Male	5	111	1
	Female	3	59	
Tumor location	Rb	5	77	0.473
	Other	3	93	
Pathological T category	≥ T3	8	104	0.027
	< T3	0	66	
Lymph node metastasis	Present	6	62	0.055
	Absent	2	108	
Pelvic lavage CEA mRNA	Positive	3	4	0.002
	Negative	5	166	
CRM status	Positive	4	7	< 0.001
	Negative	4	163	
Both CEA mRNA and CRM	Positive	3	0	< 0.001
	Negative	5	170	
Surgical approach	Open	2	11	0.107
	Laparoscopic or robot‐assisted	6	159	
Lateral pelvic lymph node dissection	Yes	4	33	0.059
	No	4	137	

### 3.7 Kaplan–Meier Analysis for Local Recurrence‐Free Survival

Kaplan–Meier analysis showed significantly worse local recurrence‐free survival in the CEA mRNA–positive and CRM‐positive groups (log‐rank test: both *p* < 0.001) (Figure 2).

**FIGURE 2:**
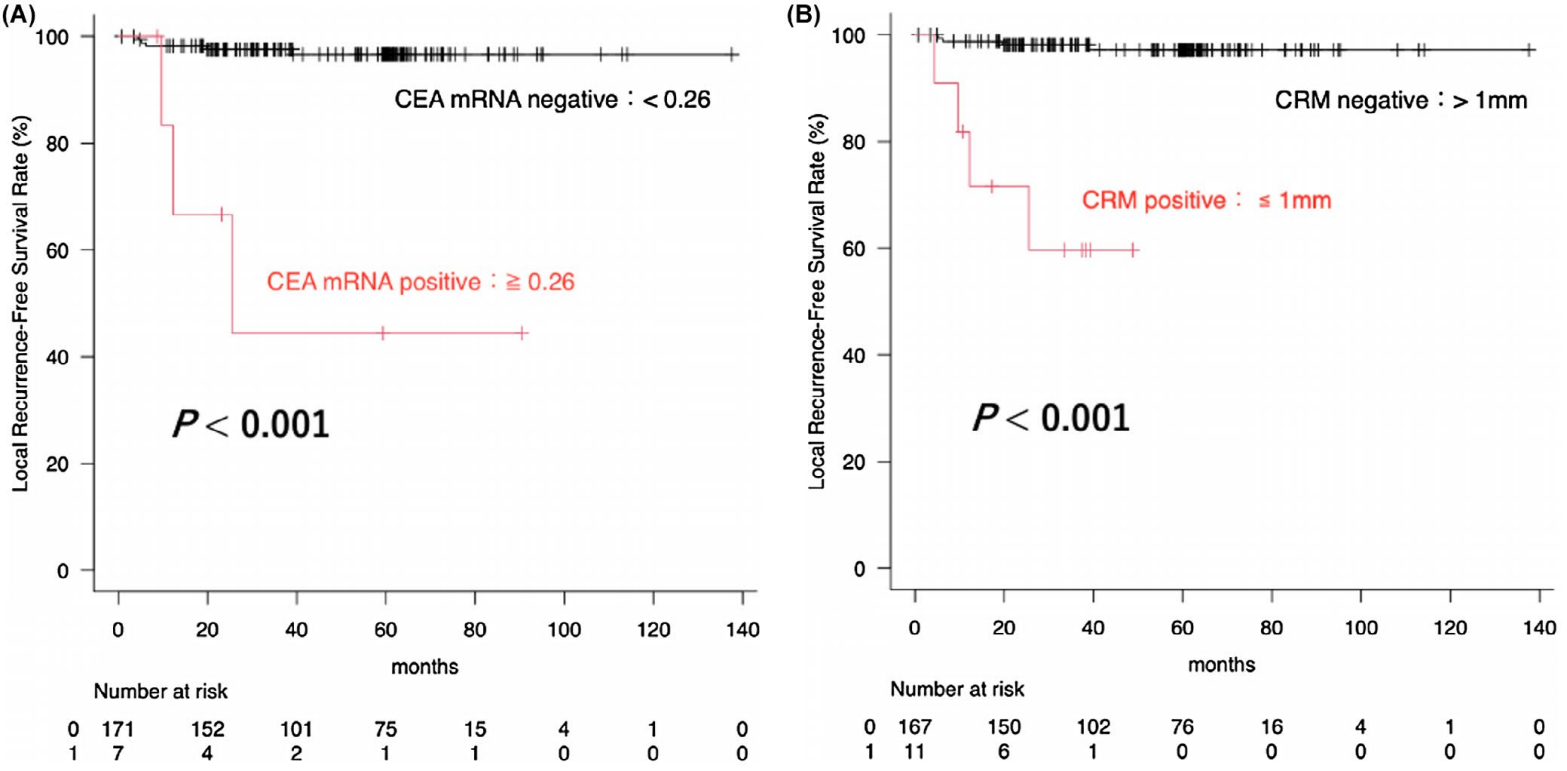
Kaplan–Meier curves for local recurrence‐free survival. (A) Local recurrence‐free survival according to CEA mRNA status. Patients with CEA mRNA positivity had significantly worse local recurrence‐free survival than those with CEA mRNA negativity. (B) Local recurrence‐free survival according to circumferential resection margin status. Patients with CRM positivity had significantly worse local recurrence‐free survival than those with CRM negativity.

### 3.8 Multivariable Analysis

In the Cox proportional hazards model including CRM status and CEA mRNA status, CRM positivity (HR 11.09, 95% CI 2.06–59.68, *p* = 0.005) and CEA mRNA positivity (HR 5.99, 95% CI 1.06–33.81, *p* = 0.043) were independently associated with local recurrence (Table 6).

**TABLE 6:** Multivariable Cox proportional hazards model for local recurrence.

Variable	Hazard ratio (HR)	95% confidence interval (CI)	*p*
CRM positivity	11.09	2.06–59.68	0.005
CEA mRNA positivity	5.99	1.06–33.81	0.043

## 4 Discussion

In this retrospective, single‐center study of 178 patients with rectal cancer who underwent curative‐intent resection, patients with detectable levels of CEA mRNA in pelvic lavage fluid had an increased risk of local recurrence. Local recurrence occurred in 4.5% of patients during a median follow‐up of 59.1 months. Both CEA mRNA and CRM positivity were associated with a higher local recurrence rate, and patients positive for both markers showed a particularly high risk. Moreover, in multivariate analysis, both CRM and CEA mRNA status remained associated with local recurrence, suggesting that pelvic lavage CEA mRNA may provide prognostic information beyond CRM status.

The CRM is a pathological metric that reflects the shortest distance between the tumor and the circumferential margin and is known to be affected by specimen handling and measurement variability. In contrast, qRT‐PCR‐based quantification of CEA mRNA offers an objective quantitative assessment under standardized laboratory conditions. Because pelvic lavage fluid may capture shed tumor cells released into the pelvic cavity during surgical dissection, this approach could reflect aspects of local recurrence risk that are not fully captured by CRM assessment alone. The observation that CEA mRNA positivity remained associated with local recurrence after adjustment for CRM suggests that these markers may represent different oncological information.

Previous studies on gastric and colorectal cancers have reported that CEA mRNA positivity in peritoneal lavage fluid is associated with recurrence and unfavorable prognosis, including in cytology‐negative patients [7, 8, 9, 10, 11, 12, 13]. However, many of these studies focused primarily on open surgery cohorts, and evidence in minimally invasive approaches, particularly robot‐assisted surgery, has been limited. Our cohort included patients who underwent laparoscopic and robot‐assisted surgeries, and our findings support the potential clinical relevance of intraoperative molecular assessment, even in contemporary minimally invasive practice.

From a clinical perspective, CEA mRNA quantification provides intraoperative information and may allow earlier risk stratification without waiting for final pathological assessment. Although immediate intraoperative strategy changes based on this marker are not currently established, it could potentially inform postoperative management, such as intensified surveillance or consideration of adjuvant therapy, particularly when interpreted in conjunction with CRM status [17]. For example, patients with CEA mRNA positivity, especially those with concomitant CRM positivity, may be candidates for closer pelvic surveillance using imaging modalities such as pelvic MRI or more frequent follow‐up. However, the present findings are insufficient to justify escalation of adjuvant treatment based solely on pelvic lavage CEA mRNA positivity. Rather, this biomarker may contribute to postoperative risk stratification and help identify patients who could benefit from intensified surveillance or future risk‐adapted treatment strategies. This marker may also serve as an objective indicator of tumor cell shedding during surgical dissection, although this hypothesis requires further investigation. Because pelvic lavage fluid was collected after rectal mobilization, the potential influence of intraoperative manipulation on CEA mRNA detection cannot be excluded. Nevertheless, whether CEA mRNA originates from preexisting tumor shedding or is influenced by intraoperative manipulation, patients with positive CEA mRNA were more likely to develop local recurrence in the present cohort. Therefore, from a clinical perspective, pelvic lavage CEA mRNA positivity may serve as a marker of local recurrence risk regardless of the underlying mechanism. Although local recurrence was more frequent than distant recurrence among patients with positive pelvic lavage CEA mRNA, the number of recurrence events was too small to permit formal statistical comparison. Therefore, it remains uncertain whether pelvic lavage CEA mRNA preferentially reflects local tumor cell dissemination or simply represents a marker of overall tumor aggressiveness.

This study had several limitations. First, its retrospective, single‐center design may limit its generalizability. Second, the number of local recurrence events and CEA mRNA–positive cases was small, which may have affected the stability of the statistical estimates. Given the limited number of local recurrence events (*n* = 8), the event‐per‐variable ratio was low (EPV = 4) in our two‐variable Cox model (CRM and CEA mRNA status). Therefore, multivariate estimates may be unstable and subject to overfitting, as reflected by the wide confidence intervals, and should be interpreted as exploratory rather than definitive evidence of independent prognostic value. Accordingly, these findings should be regarded as hypothesis‐generating and require confirmation in larger prospective cohorts.

In our cohort, pelvic lavage CEA mRNA positivity was significantly associated with open surgical approach (Table 4). In routine clinical practice, open surgery is often selected for more advanced or technically challenging cases, such as bulky tumors, suspected adjacent organ invasion, or difficult pelvic anatomy, and may therefore serve as a surrogate marker of tumor burden and operative difficulty. Accordingly, the observed association between CEA mRNA positivity and local recurrence may reflect, at least in part, case selection and residual confounding factors rather than tumor biology alone. Because of the limited number of events, we could not fully adjust for potential confounders such as pathological T category, neoadjuvant treatment, and surgical approach; thus, advanced case bias cannot be excluded.

Third, the cut‐off value for CEA mRNA positivity (0.26) was derived from ROC curve analysis in this cohort and has not been externally validated. In addition, the discriminatory ability of CEA mRNA alone was modest, with an AUC of 0.648 and a sensitivity of 37.5%. Therefore, pelvic lavage CEA mRNA should not be interpreted as a stand‐alone screening marker for local recurrence. Rather, it may be useful as a highly specific marker to identify a subset of patients at particularly high risk, especially when combined with CRM status.

Fourth, although the median follow‐up was approximately 5 years and many local recurrences occurred within the first 2–3 years, late local recurrence can occur, and a longer follow‐up may provide additional information. Fifth, 10 patients (5.6%) had Stage IV disease. No local recurrence occurred among these patients, and none were positive for pelvic lavage CEA mRNA. Therefore, the inclusion of Stage IV cases is unlikely to have materially influenced the observed association between CEA mRNA positivity and local recurrence. Nevertheless, the biological heterogeneity of Stage IV disease should be considered when interpreting the present findings.

Finally, the study period extended from 2013 to 2023, during which treatment strategies for rectal cancer evolved, including surgical approaches, neoadjuvant therapy, and postoperative adjuvant chemotherapy. Therefore, potential time‐period bias cannot be fully excluded.

Future studies should validate these findings in multicenter cohorts and assess the reproducibility and standardization of cut‐off values. Combining additional molecular markers, such as cytokeratin 20 (CK20), with CEA mRNA may improve predictive accuracy. The development of a rapid intraoperative assay could further enhance clinical utility if real‐time risk assessment becomes feasible.

## 5 Conclusions

In rectal cancer, intraoperative CEA mRNA quantification in the pelvic lavage fluid was associated with local recurrence beyond CRM status. This objective and quantitative intraoperative marker may help to stratify the risk of local recurrence and support postoperative management strategies.

## Author Contributions

**Hajime Ushigome:** investigation. **Kazuyoshi Shiga:** conceptualization, methodology, supervision, writing – review and editing. **Shuhei Uehara:** investigation. **Yoichi Matsuo:** supervision, writing – review and editing. **Hiroyuki Asai:** conceptualization, data curation, formal analysis, investigation, methodology, visualization, writing – original draft. **Takuya Suzuki:** investigation. **Hiroki Takahashi:** investigation. **Yushi Yamakawa:** conceptualization, methodology, supervision, writing – review and editing. **Akira Kato:** investigation. **Shuji Takiguchi:** supervision, writing – review and editing, project administration.

## Funding

The authors have nothing to report.

## Ethics Statement

Approval of the research protocol: This study was approved by the Institutional Review Board of Nagoya City University Hospital (approval no. 60‐19‐0150). The study was conducted in accordance with the Declaration of Helsinki.

## Conflicts of Interest

The authors declare no conflicts of interest.

## Data Availability

The data that support the findings of this study are available on request from the corresponding author. The data are not publicly available due to privacy or ethical restrictions.
